# Burden of Cancer and Utilization of Local Surgical Treatment Services in Rural Hospitals of Ethiopia: A Retrospective Assessment from 2014 to 2019

**DOI:** 10.1093/oncolo/oyac127

**Published:** 2022-07-06

**Authors:** Abigiya Wondimagegnehu, Fekadu Negash Bereded, Mathewos Assefa, Solomon Teferra, Bradley Zebrack, Adamu Addissie, Eva J Kantelhardt

**Affiliations:** Department of Preventive Medicine, School of Public Health, College of Health Sciences, Addis Ababa University, Addis Ababa, Ethiopia; Institute of Medical Epidemiology, Biometrics and Informatics, Martin-Luther-University, Halle, Saale, Germany; Department of Surgery, St Paul’s Hospital Millennium Medical College, Addis Ababa, Ethiopia; Department of Oncology, School of Medicine, College of Health Sciences, Addis Ababa University, Addis Ababa, Ethiopia; Department of Psychiatry, School of Medicine, College of Health Sciences, Addis Ababa University, Addis Ababa, Ethiopia; University of Michigan, School of Social Work, Ann Arbor, MI, USA; Department of Preventive Medicine, School of Public Health, College of Health Sciences, Addis Ababa University, Addis Ababa, Ethiopia; Institute of Medical Epidemiology, Biometrics and Informatics, Martin-Luther-University, Halle, Saale, Germany; Institute of Medical Epidemiology, Biometrics and Informatics, Martin-Luther-University, Halle, Saale, Germany; Department of Gynaecology, Martin-Luther-University, Halle (Saale), Germany

**Keywords:** cancer, rural hospitals, treatment pattern, surgical services, Ethiopia

## Abstract

**Background:**

Global cancer estimations for Ethiopia announced 77 352 new cases in 2020 based on the only population-based registry in Addis Ababa. This study characterizes cancer patients in rural Ethiopia at 8 primary and secondary hospitals between 2014 and 2019.

**Patients and Methods:**

All clinically or pathologically confirmed cancer cases that were diagnosed between 1 May 2014 and 29 April 2019 were included. A structured data extraction tool was used to retrospectively review patients’ charts and descriptive analysis was done.

**Results:**

A total of 1298 cancer cases were identified, of which three-fourths were females with a median age of 42 years. Breast (38%) and cervical (29%) cancers were the most common among females, while prostate (19%) and oesophageal cancers (16%) were the most common among males. Only 39% of tumors were pathologically confirmed. Nearly two-thirds of the cases were diagnosed at an advanced stage. Surgery was the only accessible treatment option for more than half of the cancer patients, and systemic treatment (except endocrine) was rarely available. One in 5 patients did not receive the recommended surgical procedure, half due to patient refusal or lack of the patient returning to the hospital.

**Conclusion:**

The pattern of cancer diagnoses in rural hospitals shows an exceptionally high burden in women in their middle-ages due to breast and cervical cancers. Advanced stage presentation, lack of pathology services, and unavailability of most systemic treatment options were common. The surgery was offered to nearly 60% of the patients, showing the significant efforts of health workers to reduce sufferings.

## Teaser

This article reports on the magnitude and characteristics of frequently diagnosed cancers in Ethiopia, focusing on the proportion of patients who refused surgical procedures in rural hospitals from 2014 to 2019.

**Implications for Practice**

In rural hospitals of southern Ethiopia, two-thirds of cancer patients are women in their 40s with late-stage breast or cervical malignancies. Surgery is the only treatment available and offered to nearly two-thirds of all patients, while endocrine treatment is the only systemic option available. High refusal rates for surgery may reflect the perception of cancer as a “death sentence” due to the lack of cancer survivors who encourage others to utilize oncology services early. Focusing on improving such basic services, involving the surgeons, and offering referral pathways for these patients can be the first steps to integrating oncology services into the lower levels of the health care system.

## Introduction

Cancer accounted for almost 10.0 million deaths in 2020^1^ and is projected to cause 16.2 million deaths by 2040.^2^ More than half of the reported deaths occurred in low- and middle-income countries (LMICs); particularly, Africa has a high proportion of cancer deaths (7.2% of global cancer deaths) compared with its incidence (5.7% of global cancer incidence).^2,3^ The majority of cancer patients in LMICs present at an advanced stage of the disease, which contributes to a poor prognosis of the disease and a high mortality rate.^4-6^

In Ethiopia, cancer is becoming a frequently diagnosed disease with a considerably high mortality rate. The increased incidence is due to aging and lifestyle changes. According to the Global Cancer Incidence, Mortality and Prevalence (GLOBOCAN) 2020 report, an estimated 77 352 new cases, and over 51 865 deaths were reported in the country, with the majority (50 598; 65.4%) occurring in females.^7^ During 2012-2013, a total of 4139 newly diagnosed cases were recorded in the population-based cancer registry of Addis Ababa, with age-standardized rates per 100 000 inhabitants of 136 for females and 70 for males.^8^ Similar to other LMICs, there are limited diagnostic and treatment services in Ethiopia, and the three-tier health system in the country positions oncologic care mainly at tertiary referral centers. In fact, the number of specialists and essential equipment for cancer care in Ethiopia is among the most limited and constricted in the world. Therefore, the only available service for most cancer patients at lower-level hospitals is surgery.^9,10^ Systemic treatment is limited mainly to the only cancer center in Addis Ababa and a few facilities at peripheral university hospitals. However, many cancer patients do not even receive basic surgical services. This could either be due to a lack of surgical capacity or organizational problems. On the other hand, the patients may not be psychologically ready to receive the necessary surgical procedures with perceived unacceptable bodily mutilation or fear of systemic therapy for unknown reasons. As a result, these patients may seek help from alternative healers, such as herbalists, priests, and spiritual leaders,^11,12^and return to health facilities once complications and advanced-stage cancer occur.^13-15^

Even though there are studies about cancer care in Ethiopia, cancer statistics are either limited to tertiary level hospitals or only in the capital city where the cancer registry is based. However, to the best of our knowledge, the magnitude of cancer patients and reasons for non-uptake of surgical and other treatment options are not well documented in Ethiopia, particularly at lower-level rural hospitals. Therefore, this study is intended to determine the magnitude and characterize frequently diagnosed cancers, describe the uptake of surgical services, and calculate the proportion of patients who refused surgical procedures in rural hospitals in Ethiopia from 2014 to 2019.

## Materials and Methods

### Study Design and Area

The study was conducted in 8 rural hospitals in southern and southwestern Ethiopia: 6 secondary level hospitals—St. Lukas Catholic Hospital, Butajira General Hospital, Dubo St. Mary Hospital, Negist Elleni Hospital, Durame General Hospital, and Wollayta Teaching and Referral Hospital; and 2 primary level hospitals—Attat our First Lady of Lourdes Catholic Primary Hospital and Aira General Hospital. The majority (6) of them are located in the Southern Nations, Nationalities, and Peoples’ Region (SNNPR), while St. Lukas Catholic Hospital and Aira General Hospital are situated in the Oromia region. These hospitals were chosen based on a preliminary assessment of their ability to provide cancer diagnostic and treatment services, as well as their relatively large rural catchment populations of 0.8-2.5 million people. The hospitals have 4 major inpatient departments (medical, surgical, pediatric, and gynecology/obstetrics) with a total of 100-350 beds. Diagnostic services such as common laboratory tests, X-ray and ultrasound are widely available in these hospitals. However, other imaging services such as computed tomography (CT scan) and magnetic resonance imaging (MRI) and pathologic tests like fine needle aspiration cytology (FNAC) and biopsy are provided through referral linkage with higher-level hospitals in Addis Ababa. A 5-year retrospective patient file data review was done from May 2019 to June 2019.

### Study Participants

All clinically certain and/or pathologically confirmed cancer cases who were above 18 years old and who were diagnosed and possibly treated in those 8 hospitals between 1 May 2014 and 29 April 2019 were included.

### Data Collection and Analysis

The diagnoses and treatments of patients at the included hospitals within the study period were ascertained from the admissions and outpatient logbooks of the hospitals. Subsequently, the cards of patients with an assessment of any type of cancer or suspected cancer were extracted from the database using the medical registration number. A structured data extraction tool was prepared based on the objective of the study and important variables, such as sociodemographic characteristics, clinical features, and type of treatment received (including reasons for non-uptake) were included. Eight health management information system (HMIS) focal persons who were working in those hospitals were involved in screening the logbooks and tracing all the files of patients who were diagnosed and treated in the past 5 years. A total of 16 data collectors (2 per site) who had BSc degrees in nursing were recruited to extract all the important information from the cards of patients. All collected data forms were assessed for data quality, completeness, and consistency and descriptive statistics were done using SPSS software version 25.

### Ethical Clearance

Ethical clearance was obtained from the Addis Ababa University, College of Health Sciences, Institutional Review Board. Individual informed consent was waived due to the retrospective nature of the study.

## Result

### Sociodemographic Characteristics

During the study period, a total of 1511 cancer cases were identified in the 8 rural hospitals. Of those, the actual diagnosis was neither confirmed pathologically nor clinically for 213 (14.1%) cases, and no additional information was recorded for these cases, except stating that to “rule out a specific cancer.” Therefore, a total of 1298 cancer patients either clinically or pathologically confirmed were included in our final analysis. The median age of participants was 42 years, with an interquartile range of 17 years. Three-fourths of all cases were females and more than half (52.9%) of them were married. Regarding their religious views, one-quarter (25.3%) of them were Protestants, while nearly one-fifth (17.3%) of them were Orthodox Christians. Even though educational status was not recorded for almost half (49%) of the cases, one-quarter were known to be illiterate and only one-tenth had attended formal education. More than one-third (35.5%) of cases were housewives and one-eighth were farmers. The majority of cases were identified at St. Lukas Catholic Hospital and Aira General Hospital, each accounting for one-fifth of the total cases (Table 1).

**Table 1.** Sociodemographic characteristics of cancer patients diagnosed in 8 rural hospitals of Ethiopia between 2014 and 2019.

Variables	*n* = 1, 298	Percent (%)
Sex		
Male	304	23.4
Female	983	75.7
Not recorded	11	0.8
Age		
18-29	167	12.9
30-44	520	40.1
45-59	386	29.7
60-74	189	14.6
>75	36	2.8
Name of the hospitals (catchment population in million)		
St Lukas (1.3)	267	20.6
Aira^+^(1.3)	254	19.6
Durame (1)	156	12.0
Atat^+^ (0.8)	150	11.6
Negest Elleni (2.5)	136	10.5
Ottana (2)	135	10.4
Dubo (0.8)	110	8.5
Butajira (1.3)	90	6.9
Origin of patients (region)		
Oromia	470	36.2
SNNPR	800	61.6
Others^*^	4	0.3
Not recorded	24	1.8
Religion		
Orthodox	224	17.3
Muslim	148	11.4
Protestant	328	25.3
Catholic and traditional	5	.4
Not recorded	593	45.7
Education		
Illiterate	331	25.5
Read and write	191	14.7
Primary education	72	5.5
Secondary education	40	3.1
Higher education	28	2.2
Not recorded	636	49.0
Marital status		
Single	60	4.6
Married	686	52.9
Divorced/separated	18	1.4
Widowed	31	2.4
Not recorded	503	38.8
Occupation		
Housewife	461	35.5
Farmer	176	13.6
Civil servant	21	1.6
Student	38	2.9
Others ^a^	19	1.5
Not recorded	583	44.9

Primary hospitals.

Others include Amhara and Addis Ababa.

Others include factory, merchant, and private.

Abbreviation: M, million.

**Figure 1.**
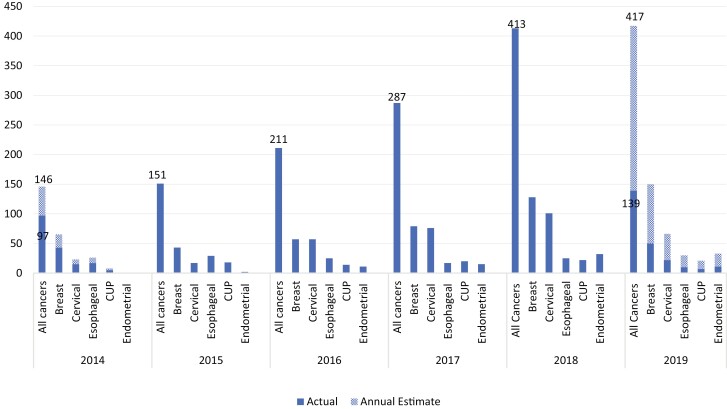
Types of cancer cases diagnosed in 8 rural hospitals of Ethiopia from 2014 to 2019 (*n* =1298). Actual data was collected for 8 complete months in 2014 and 4 months in 2019. Based on that, it’s extrapolated for the whole year.

Concerning the distribution of cases over the past 5 years, the number of cancer cases has been persistently increasing throughout these years from 97 (7.5%) cases in 2014 to 409 (31.8%) cases in 2018. Even though the data collected in 2014 was only for 8 months, the total cases reported was still far lower compared to the other years. In 2019, a total of 139 (10.7%) cases were identified from January to April, suggesting that the total number of cases at the end of the year might even be higher than the previous year (Fig 1).

### Types of Cancer

Out of the total 1298 cancer patients who visited those 8 hospitals between 2014 and 2019, almost one-third of them were diagnosed with breast cancer. Cervical cancer was the second most common type of cancer, accounting for 22% of total cases. Oesophageal (9.5%), endometrial (5.5%), colorectal (5.2%), gastric (4.9%), and prostate (4.5%) were the other frequently diagnosed cancers during the stated period. The five most common cancers among females were breast, cervical, oesophageal, endometrial, and gastric, while prostate was the most common cancer among males, followed by oesophageal cancer, colorectal cancer, cancer of unknown origin (CUP), and gastric cancer (Table 2). Breast cancer was leading cancer in all rural hospitals, except for St. Lukas Catholic Hospital and Aira General Hospital (Table 3).

**Table 2.** Types of cancer diagnosed in men and women.

Primary site of cancer	Males *n* (%)	Females *n* (%)	Unknown *n* (%)	Total *n* (%)	Pathologically confirmed *n* = 503
Breast	23 (7.6)	372 (37.8)	5 (45.5)	400 (30.8)	221 (55.3)
Cervical	0 (0)	289 (29.4)		289 (22.3)	141 (48.8)
Esophageal	49 (16.1)	74 (7.5)		123 (9.5)	5 (4.1)
CUP	37 (12.2)	46 (4.7)	3 (27.3)	86 (6.6)	24 (27.9)
Endometrial	0 (0)	71 (7.2)		71 (5.5)	30 (42.3)
Colorectal	45 (14.8)	19 (1.9)		67 (5.2)	12 (18.8)
Gastric	33 (10.9)	33 (3.4)	1 (9.1)	64 (4.9)	9 (13.4)
Prostate	58 (19.1)	0 (0)		58 (4.5)	15 (25.9)
Ovarian	0 (0)	30 (3.1)		30 (2.3)	11 (36.7)
Soft tissue	9 (3.0)	15 (1.5)	1 (9.1)	24 (1.8)	13 (54.2)
Bladder	10 (3.3)	2 (0.2)		13 (1.0)	0 (0)
Liver	7 (2.3)	3 (0.3)		10 (0.8)	1 (10.0)
Lung	3 (1.0)	5 (0.5)	1 (9.1)	9 (0.7)	2 (22.2)
Hematologic	3 (1.0)	5 (0.5)		8 (0.6)	0 (0)
Testicular	7 (2.3)	0 (0)		7 (0.5)	1 (14.3)
Thyroid	1 (0.3)	7 (0.7)		8 (0.6)	8 (100.0)
Pancreatic	3 (1.0)	2 (0.2)		5 (0.4)	2 (40.0)
Others*	16 (5.3)	10 (1.0)		26 (2.0)	8 (30.8)
Total	304 (100)	983 (100)	11 (100)	1298 (100)	503 (38.8)

Others: include inguinal, cholangial, renal, laryngeal, vulvar, penis, skin, and tongue cancer.

Abbreviation: CUP, cancer of unknown origin.

**Table 3.** Five top cancers diagnosed in 8 rural hospitals of Ethiopia between 2014 and 2019.

Top 5 cancers	Name of the hospitals							
	SNNPR region Primary and secondary level hospitals						Oromia region primary level hospitals	
	Attat (150)	Negest E. (136)	Dubo(110)	Durame (156)	Ottana (135)	Butajira (90)	St. Lukas (267)	Aira (254)
1^st^	Breast (84)	Breast (52)	Breast (33)	Breast (51)	Breast (60)	Breast (17)	Cervical (99)	Esophageal (79)
2^nd^	CUP (43)	Cervical (44)	CUP (22)	Cervical (17)	Cervical (32)	CUP (15)	Endometrial (38)	Breast (69)
3^rd^	Esophageal (10)	Gastric (9)	Cervical (14)	Prostate (17)	Colorectal (10)	Gastric (13)	Breast (34)	Cervical (68)
4^th^	Cervical (5)	Colorectal (8)	Endometrial (13)	Gastric (16)	Soft Tissue (6)	Esophageal (12)	Prostate (17)	Colorectal (12)
5^th^	Prostate (5)	Ovarian (6)	Gastric (10)	Esophageal (15)	Gastric (6)	Cervical (10)	Ovarian (6)	Endometrial (11)

Note: the values in bracket are actual number of cases.

Abbreviation: CUP, cancer of unknown primary.

### Medical and Clinical Characteristics

In this study, most (785; 60.5%) of the cases were diagnosed clinically, while only 503 (38.8%) of the cases were pathologically confirmed. Out of those cases with pathological confirmation, the histologic type was not recorded for 41.6%. Squamous cell carcinoma (44.6%) was the most common type of cancer, of which 80.9% were reported among cervical cancer patients, while ductal cell carcinoma (25.2%) and adenocarcinoma (12.2%) were the second and third common types of cancer with known histology, respectively. Out of the total of pathologically confirmed cancer cases, tumor grade was recorded only for 144 (28.6%) cases, of which 13.9% were grade III and 9.7% were grade II cancers. Concerning the stage of cancer, nearly two-thirds (65.4%) of the patients with a known stage were diagnosed at an advanced stage of cancer, either at stage III (37.4%) or stage IV (28.0%) (Table 4).

**Table 4.** Medical and clinical characteristics of participants.

Variables	*n* = 1, 298	Percent (%)
Method of diagnosis		
Clinically	785	60.4
Pathologically	503	38.8
Not recorded	10	0.8
Histologic type (*n* = 294)		
Squamouscell carcinoma	131	44.6
Ductal cell carcinoma	74	25.2
Adeno carcinoma	36	12.2
Adeno squamous cell carcinoma	21	7.1
Other types^*^	32	10.9
Tumor grade (*n* = 144)		
Grade I	25	17.4
Grade II	49	34.0
Grade III	70	48.6
Stage (*n* = 364)		
Stage I	16	4.4
Stage II	110	30.2
Stage III	136	37.4
Stage IV	102	28.0
Why surgery not planned (*n* = 519)		
Advanced cancer	18	3.5
Referred	350	67.4
Not recorded	151	29.1
Why surgery was not done (*n* = 140)		
Refused/patient did not return back	69	49.3
Planned in the future	16	11.4
Referred	45	32.1
Other reasons ^c^	10	7.1
Hormonal treatment for breast cancer (*n* = 400)		
Yes	119	29.8
No	258	64.5
Not recorded	23	5.7
Duration of hormonal treatment (*n* = 119)		
1-3 months	27	22.7
4-6 months	17	14.3
7-9 months	6	5.0
10-12 months	15	12.6
>1 year	20	16.8
Not recorded	34	28.6
Chemotherapy		
Yes	18	1.4
No	1,062	81.8
Not recorded	218	16.8
Radiotherapy		
Yes	9	0.7
No	1,058	81.5
Not recorded	231	17.8
Total patients referred (*n* = 429)		
Immediately after diagnosis	350	81.6
After surgery planned	45	10.5
For further treatment	34	7.9
Name of health facility referred (*n* = 429)		
Tikur Anbessa specialized hospital	214	49.9
Hospital in Addis Ababa	36	8.4
Hawassa referral hospital	29	6.8
Hossana referral hospital	20	4.7
Wolayta teaching and referral hospital	11	2.6
Other hospitals^b^	3	0.7
Not recorded	116	27.0
Patient status mentioned in the patient file		
Died in hospital	73	5.6
Not recorded	1228	94.4

Other types include—lobular, papillary, and basal cell carcinoma.

Other reasons include poor medical condition, respiratory distress, and to resuscitate patients.

Other hospitals include—Durame and Nekemete hospitals

### Treatment Options and Outcome

**Figure 2.**
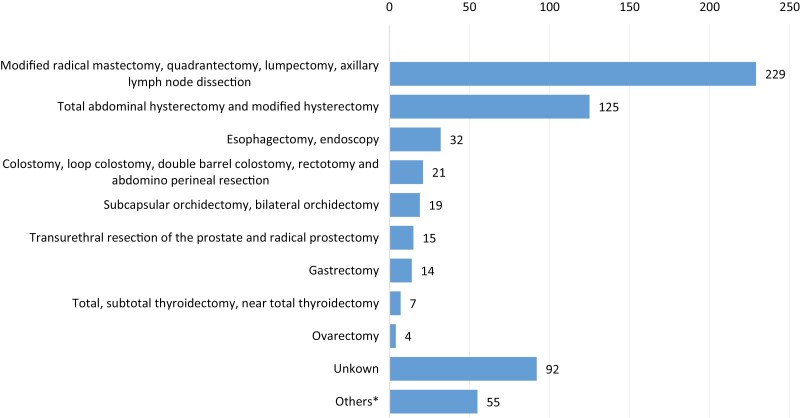
Types of surgery done for cancer patients in 8 rural hospitals of Ethiopia from 2014 to 2019 (*n* = 612). *Others**—**include laparotomy, local excision, tracheostomy, amputation and split-thickness skin grafting (STSG).

Surgical interventions were the most common cancer treatments in those rural hospitals, and surgery was planned for more than half (756; 58.2%) of the total cases. The major reasons why surgery was not planned for the remaining (519; 40.0%) cases were as follows: firstly, patients were directly referred to the next level health facility for further investigations (350; 67.4%) and secondly, patients with advanced cancer at the time of diagnosis (18; 3.5%) were not operated on. Out of a total of 756 planned surgical procedures, the majority (81.0%) were performed in those hospitals, while the rest (18.5%) were not done due to several factors. One of the major reasons was either patient refusal, or patients’ not returning to the hospital after having an appointment. This accounted for almost half (49.3%) of the planned procedures. The rest of the patients were referred to the next level (32.1%) or planned in the future at the time of data collection (11.4%) (Table 4). With regard to the type of surgery, mastectomy was the most frequently performed surgical procedure, concurrent with the high prevalence of breast cancer in those hospitals. Abdominal hysterectomy was the second most common surgical procedure, which accounted for 20.4% of the total surgeries performed during the study period (Fig 2). Out of the total of 612 surgeries performed in those hospitals, the intent of the surgery was mentioned to be curative for only 12.1% of the patients, whereas palliative surgery was performed for almost two-thirds (66.3%) of the patients. However, the intention of the surgery was not specified for the remaining 21.6% of patients (Table 5).

**Table 5.** Site of tumor and intention of surgery.

Primary site of cancer	Surgery planned *n* = 756 (% of total within entities)	Surgery performed *n* = 612 (% of total within entities)	Curative surgery *n* = 74 (% of total within entities)	Palliative surgery *n* = 406 (% of total within entities)	Intent not specified *n* = 132 (% of total within entities)
Breast	319 (79.8)	271 (85.0)	30 (11.1)	198 (73.1)	43 (15.9)
Cervical	96 (33.2)	82 (85.4)	10 (12.2)	67 (81.7)	5 (6.1)
Esophageal	47 (38.2)	38 (83.0)	0 (0)	3 (7.7)	36 (92.3)
CUP	46 (53.5)	29 (63.0)	3 (10.3)	16 (55.2)	10 (34.5)
Endometrial	57 (80.3)	50 (87.7)	19 (38.0)	27 (54.0)	4 (8.0)
Colorectal	39 (60.9)	32 (82.1)	2 (6.3)	23 (71.9)	7 (21.9)
Gastric	39 (58.2)	20 (51.3)	0 (0)	13 (65.0)	7 (35.0)
Prostate	47 (81.0)	41 (87.2)	3 (7.3)	29 (70.7)	9 (22.0)
Ovarian	18 (60.0)	16 (88.9)	1 (6.3)	14 (87.5)	1 (6.3)
Others*	14 (53.8)	10 (71.4)	2 (20.0)	5 (50.0)	3 (30.0)
Soft tissue	14 (53.3)	11 (78.6)	3 (27.3)	3 (27.3)	5 (45.5)
Bladder	6 (46.2)	1 (16.7)	0 (0)	0 (0)	1 (100)
Liver	2 (20.0)	2 (100)	0 (0)	2 (100.0)	0 (0)
Lung	2 (22.2)	0 (0)	NA	NA	NA
Hematologic	0 (0)	NA	NA	NA	NA
Testicular	4 (57.1)	2 (50.0)	0 (0)	1 (50.0)	1 (50.0)
Thyroid	6 (75.0)	6 (100)	1 (16.7)	5 (83.3)	0 (0)
Pancreatic	0 (0)	NA	NA	NA	NA
Total	756 (58.2)	612 (81.2)	74 (12.1)	406 (66.3)	132 (21.6)

*Others include inguinal, cholangial, renal, laryngeal, vulvar, penis, skin, and tongue cancer.

Abbreviation: CUP, cancer of unknown primary.

Concerning other types of cancer treatment, hormonal therapy was given to almost one-third (29.8%) of breast cancer patients. Out of these, 83.2% of them were taking tamoxifen; the type of drug was not recorded for the remaining 16.8% of cases. More than half (54.6%) of these patients were taking hormonal treatment for less than a year. However, none of the prostate cancer patients received hormonal treatment. According to the medical records, chemotherapy and radiotherapy (RT) services were not available in those hospitals, and only 1.4% and 0.7% of the total patients reported having ever received chemotherapy and RT, respectively (Table 4). It’s also indicated that they have received these services at Tikur Anbessa Specialized Hospital, which is the largest hospital providing comprehensive cancer care in Ethiopia.

In relation to the referral system, around one-third (33.1%) of total cancer patients were referred to the next high-level health facility for either of the following reasons: immediately after diagnosis for additional investigations or for further systemic treatments such as chemotherapy and RT after receiving surgical treatment. Even though the exact referral sites were not recorded for 27.0% of these patients, nearly half (49.9%) of them were referred to the comprehensive cancer center in Addis Ababa. Based on the information documented in the charts of patients, 5.6% of in-hospital deaths were recorded, while the status of the remaining 94.4% of cases was unknown at the time of data collection (Table 4).

## Discussion

This study described the overall burden of cancer cases in 8 rural hospitals in Ethiopia from 2014 to 2019. Our results revealed that the number of diagnosed cancer cases had been remarkably increasing over the years, and three-fourths of the cases were reported among females. Breast and cervical cancers were the most frequently diagnosed cancers among females, while prostate and oesophageal cancers were the most frequently diagnosed cancers among males. The majority of the cancer cases were diagnosed clinically, and pathologic evaluations were done only for one-third of the total cases.

More than 80% of the cancer cases in this study were in individuals below the age of 60 years. This finding is consistent with the population-based cancer registry in Addis Ababa, which stated that 72% of total cancer cases diagnosed from 2012 to 2014 were below the age of 60 years.^8^ According to a recent study in 2020, 65% of total cases were below the age of 60 years,^16^ and others reported that the median age of cancer patients ranged from 48 to 52 years.^12-14^ Not only in Ethiopia but also in several LMICS, cancer is more common among individuals below 65 years of age.^17,18^ This variation is mainly due to the young population structure in Ethiopia and in most low-income countries.

In this study, three-fourths (75.7%) of the total cancer cases were females, and a similar (72.8%) figure was reported from a record review done at the only oncology center in the country from 1998 to 2010.^19^ Previous hospital-based and population-based studies also stated that more than 60% of cancer cases in Ethiopia occur among females.^8,13,16^ This can be explained by the fact that breast and cervical cancers are the 2 most frequently diagnosed cancers occurring mainly/only in females. Our study also confirms that, in rural Ethiopia, cancer is a diagnosis in women who are very young, often have children at an early age, and are responsible for looking after their children (who likewise suffer).^20^

Our results suggest that the number of cancer cases presenting at hospitals is increasing over time, up to 4 times, between 2014 and 2018. This reflects the aging of the population, an increase in service uptake, and possibly lifestyle changes. Our findings support the global burden of cancer increases over time and is projected to be 29.5 million over the next 2 decades,^4^ and by 2035, it’s estimated that two-thirds of these new cancer diagnoses will occur in developing countries.^2^

Our results showed that breast and cervical cancers were the 2 leading cancers for both sexes, each accounting for 30.8% and 22.3% of total cancer cases, again showing the huge burden for women. Similar figures were reported from 2013 Addis Ababa population-based cancer registry data, in which 31.4% and 14.1% of total cancer cases were due to breast and cervical cancers, respectively.^8^ In the tertiary cancer center in Addis Ababa, gynaecological malignancies (predominantly cancer of the uterine cervix) were found to be leading cancer, followed by breast cancer based on the retrospective review done from 1998 to 2010.^19^ The University of Gondor Hospital also reported that cervical cancer was the most common cancer, accounting for 16.1% of total cases diagnosed and treated within a 1-year duration.^21^ This might be due to the unavailability of RT services at lower-level hospitals and all cervical cancer cases ending up in a tertiary hospital, whereas breast cancer cases can receive surgical and other treatment options at primary or secondary level hospitals. In summary, these 2 cancers are the most prominent cancers in tertiary hospitals, in lower-level hospitals and also among the population, accounting for the lion’s share of cancer deaths among females. The discrepancy in cervical cancer incidence and mortality between developed and developing nations is striking.^1,3^ In 2020, Africa contributed to 19.4% of all incident cases worldwide,^1^ and East Africa has the highest subregional incidence of cervical cancer.^5,22^ This tragedy is due to a lack of screening services and human papillomavirus (HPV) vaccination in the region.

During the stated period, prostate (19.1%) cancer was the first cancer among males, followed by oesophageal (16.1%) and colorectal (14.8%) cancers. This differs slightly compared to findings in the Addis Ababa population-based cancer registry, showing that the three most common cancers among males were colorectal, non-Hodgkin’s lymphoma, and prostate cancer.^23^ However, leukaemia and non-Hodgkin’s lymphoma were rarely found in our study. The main reason for this might be the lack of advanced diagnostic services and specialists, and thus a lack of detection of these cases in lower-level hospitals. The other possible explanation for this could be physicians considering such cases as HIV or other diseases and never suspecting a malignancy.

Out of the total 1298 cancer cases identified in our study; oesophageal cancer (9.5%) was the third most common cancer. Previous studies from other geographic areas reported it as the ninth most incident and the eighth leading cause of mortality in Ethiopia, contributing to less than 3% of total cases.^8,13,16,23^ We found Aira General Hospital to be a hotspot in our study, and it is located in West Wellega zone of the Oromia region. Out of the total of 123 oesophageal cases reported within a 5-year duration, nearly two-thirds (79; 64.2%) were reported from this hospital. Several studies conducted in Ethiopia also revealed certain hotspots in the Oromia region, particularly in the Arsi and West Wellega zones.^24-26^ Consumption of hot beverages and porridge in these places has recently been identified as a risk factor for oesophageal cancer, along with other environmental factors.^27,28^

The majority of our cases were diagnosed clinically, and pathologic examination was done only for one-third (38.8%) of total cases, of which the histologic type and tumour grade were recorded only for 209 (41.6%) and 144 (28.6%) of cases, respectively. Several studies both in Ethiopia and other LMICs have highlighted that the majority of cancer cases were often diagnosed and treated clinically without verification through biopsy or other pathologic examination.^4,6,12^ In Ethiopia, health facilities with FNAC and biopsy services are rare and mainly concentrated in urban areas. Therefore, patients are forced to travel long distances and spend a lot of money, especially for private services. Lack of pathology has many implications in terms of the provision of standard cancer care relying on histologic type, grade, and immunohistochemistry for breast cancer. The unavailability of local pathologic services is one of the main contributing factors to the late diagnosis of cancer in Ethiopia, together with weak referral systems and extended waiting periods for consultation.^11,29,30^

In LMICs, the majority of cancer patients are diagnosed at an advanced stage of the disease, which contributes to a high mortality rate and poor prognosis of the disease.^4-6,17^ In this study, nearly two-thirds (238; 65.4%) of the total cases were diagnosed at an advanced stage of the disease. The majority of Ethiopian studies found that more than 70% of cancer cases in Ethiopia were diagnosed at stages III and IV.^13,14,16,23,25,31^ According to a multicentre study in southern Ethiopia, 72.5% of breast cancer patients were diagnosed at an advanced stage of the disease.^29^ Age above 40 years, lower educational levels, rural residence, visiting traditional healers and religious places, lack of awareness about cancer (including its risk factors), screening techniques, and severity of the disease were some of the identified factors associated with late-stage presentation.^6,12,30,31^ Moreover, negative perceptions toward medical treatment,^23,32^ low screening service uptake, and poor health-seeking behaviors^32-34^were the other reasons for the late presentation of cancer in the country.

Surgery is essential for global cancer care in all resource settings.^35,36^ It’s shown to be a vital method in the management of most patients with cancer.^37^ Out of the 15.2 million new cases of cancer in 2015, over 80% of individuals with cancer required a surgical procedure, yet only 25% of cancer patients worldwide and less than 5% in low-income countries get timely, affordable, and safe surgery.^35^ Our results showed that surgery is the most widely available cancer treatment option in the primary and secondary rural hospitals of Ethiopia, since systemic treatments, such as chemotherapy and RT services, are scarcely available at tertiary level hospitals. Studies also reported that, for most patients with cancer in Africa, surgery alone is the only realistically available option because of disorganized referral and treatment patterns.^37^ Additionally, in many cases, the cost of neoadjuvant chemotherapy and RT is 2 to 3 times higher than the cost of all surgical care.^35,36^

Mastectomy was the most frequently performed surgical procedure, followed by total abdominal hysterectomy. Modified radical mastectomy is the recommended surgical procedure for all breast cancer patients in Ethiopia due to the low capacity for RT services that would be needed for breast conservation. This might have a lot of implications in terms of refusal and discontinuation of treatment. In this study, 49.3% of planned surgical procedures were not performed because either the patient refused or did not return to the hospital for follow-up on the appointed date. The most obvious reasons for not having surgery could be financial problems,^36,37^ fear of the surgical procedure, or complications after the operation.^36^ The other possible explanation is in association with visiting traditional healers and religious places.^11,12,29,30^ In many parts of Africa, there are well-entrenched beliefs that cancers should not be touched and, furthermore, touching them through surgical resection or biopsy will lead to the acceleration of an already fatal disease. This belief frequently leads to very late presentation and refusal of surgical procedures^37^ and eventually a high mortality rate, which is a vicious cycle will confirm the belief that cancer cannot be cured anyway. In addition, the lack of any systemic treatment for the rural population by itself already leads to fatal outcomes. Endocrine treatment may be an option to task-share with oncology services at lower-level hospitals.

## Strengths and Limitations

One of the strengths of this study was the inclusion of patients from 8 primary and secondary hospitals selected from the Oromia and SNNPR regions; the 2 largest regions in the country with considerable diversity of patient characteristics and health services. Having both primary and secondary hospitals in our study also enabled us to compare the disease pattern and provision of cancer care at different health tiers. Five-year data can reflect the trend in the uptake of cancer services over the years. However, since the data were retrospectively reviewed from the charts of patients, missing data for important variables was one of the limitations of the study. In addition, there was a chance of missing the cards of patients because of poor storage of charts in those hospitals, which might have underestimated the total number of cases diagnosed and treated in these hospitals during the stated period. Due to the lack of awareness and shortage of accurate diagnostic services, certain cancers probably never reached the rural hospitals or, if they did, they were not identified clinically without advanced imaging or other services.

## Conclusion

Nearly 1300 total cancer cases were diagnosed and treated in 8 rural hospitals in Ethiopia, showing an increment over a 5-year duration. The majority of the burden of more than three-fourths of cancers was weathered by females. Breast and cervical cancers were the most common cancers among females, while prostate and oesophageal cancers ranked first and second among males, respectively. The majority of the cancer cases were diagnosed clinically, and pathologic tests were done only for one-third of the total cases, showing an urgent need for more pathology services. Given the lack of pathologists in the country, innovative strategies, such as sample collection services or telepathology, should be considered. Surgery is the predominantly available cancer treatment in these hospitals and is offered to 60% of the patients, showing the considerable efforts of the local surgeons. Of these surgeries, one-fifth were not performed due to patient refusal or patients not returning for appointments. This clearly shows the need to better understand the concepts and beliefs of patients within the community. Interdisciplinary research is required to find a way to talk about medical treatment options for rural populations and their concepts of health. Nearly none of the patients had records of receiving chemotherapy or RT at cancer centers, pointing to a lack of clear patient navigation and possibly the absence of reporting back from higher-level hospitals. In summary, we found considerable efforts by health workers, including surgeons, in remote areas to treat cancer patients with mainly late-stage diseases. Integrating oncology care into lower-level hospitals needs careful consideration for task-sharing, in addition to improving the linkage to higher-level services. Access, timeliness, quality, and affordability of the service need to be considered, and only then can the vicious circle of late-stage presentation, resulting in high mortality despite treatment and the perception of cancer as “a death sentence anyway” in the population, be broken.

## Data Availability

The datasets used and/or analyzed during the current study are available from the corresponding author on reasonable request.
